# A Cytolethal Distending Toxin Variant from *Aggregatibacter actinomycetemcomitans* with an Aberrant CdtB That Lacks the Conserved Catalytic Histidine 160

**DOI:** 10.1371/journal.pone.0159231

**Published:** 2016-07-14

**Authors:** Davor Obradović, Rok Gašperšič, Simon Caserman, Adrijana Leonardi, Maja Jamnik, Zdravko Podlesek, Katja Seme, Gregor Anderluh, Igor Križaj, Peter Maček, Matej Butala

**Affiliations:** 1 Department of Biology, Biotechnical Faculty, University of Ljubljana, Ljubljana, Slovenia; 2 Department of Oral Medicine and Periodontology, Faculty of Medicine, University of Ljubljana, Ljubljana, Slovenia; 3 Laboratory for Molecular Biology and Nanobiotechnology, National Institute of Chemistry, Ljubljana, Slovenia; 4 Department of Molecular and Biomedical Sciences, Jožef Stefan Institute, Ljubljana, Slovenia; 5 Institute of Microbiology and Immunology, Faculty of Medicine, University of Ljubljana, Ljubljana, Slovenia; 6 Department of Chemistry and Biochemistry, Faculty of Chemistry and Chemical Technology, University of Ljubljana, Ljubljana, Slovenia; Karolinska Institutet, SWEDEN

## Abstract

The periodontopathogen *Aggregatibacter actinomycetemcomitans* synthesizes several virulence factors, including cytolethal distending toxin (CDT). The active CDT holoenzyme is an AB-type tripartite genotoxin that affects eukaryotic cells. Subunits CdtA and CdtC (B-components) allow binding and intracellular translocation of the active CdtB (A-component), which elicits nuclear DNA damage. Different strains of *A*. *actinomycetemcomitans* have diverse virulence genotypes, which results in varied pathogenic potential and disease progression. Here, we identified an *A*. *actinomycetemcomitans* strain isolated from two patients with advance chronic periodontitis that has a regular *cdtABC* operon, which, however, codes for a unique, shorter, variant of the CdtB subunit. We describe the characteristics of this CdtBΔ116–188, which lacks the intact nuclear localisation signal and the catalytic histidine 160. We show that the *A*. *actinomycetemcomitans* DO15 isolate secretes CdtBΔ116–188, and that this subunit cannot form a holotoxin and is also not genotoxic if expressed ectopically in HeLa cells. Furthermore, the *A*. *actinomycetemcomitans* DO15 isolate is not toxic, nor does it induce cellular distention upon infection of co-cultivated HeLa cells. Biological significance of this deletion in the *cdtB* remains to be explained.

## Introduction

The bacterium *Aggregatibacter actinomycetemcomitans* is an important etiological agent that is implicated in the onset and progression of aggressive forms of periodontitis [1]. Upon disruption of the equilibrium of the resident oral microbiota, *A*. *actinomycetemcomitans* up-regulates several virulence traits that enable it to adhere to and colonise the host, where it can cause periodontal tissue damage [2]. Several strains of *A*. *actinomycetemcomitans* secrete a heterotrimeric AB-type cytolethal distending toxin (*Aa*CDT) [3]. Similar to other CDTs from Gram-negative bacteria, *Aa*CDT is a tripartite polypeptide toxin that is composed of subunits A, B and C (*Aa*CdtA, B, C). *Aa*CDT can elicit cell-cycle-mediated growth arrest that results in cell distention of eukaryotic cells [4], and cell death by apoptosis [5]. The CDT holotoxin, CdtABC, promotes DNA breaks in host cells that lead to activation of the DNA damage response. Chronic intoxication with sub-lethal CDT doses has been shown to elicit genomic instability [6], which is a hallmark of carcinogenesis [7].

The CdtA and CdtC subunits promote the entry into cells of the enzymatic CdtB subunit via a dynamin-dependent pathway that transports CdtB through the Golgi apparatus, into the endoplasmic reticulum, and thence to the host-cell nucleus [8, 9]. The amino-terminal segment of CdtB carries a nuclear localization signal that appears to mediate the active nuclear import of CdtB [10]. It has been generally recognized that CdtB has toxic effects via its DNase and phosphatidylinositol 3,4,5-trisphosphate phosphatase activity, which can result in chromosomal damage that leads to cell-cycle arrest, progressive cytodistention, and death of susceptible eukaryotic cells [11–13]. The conserved catalytic DNase I residues of *A*. *actinomycetemcomitans* CdtB include H160 and H274, and these appear to be essential for phosphodiester bond hydrolysis [11]. These histidines are well preserved among all CdtBs from various organisms [14].

The biology of the *Aa*CDTs is not completely understood [15, 16]. Prevalence of *Aa*CDT production in individuals with periodontitis is between 27% and 79% [17–20]. Although the reports imply that the presence of the toxin is associated with periodontitis in humans, in a rat model, CDT production by *A*. *actinomycetemcomitans* did not cause any periodontal disease, nor bone loss [21].

Genetically distinct clonal lineages of *A*. *actinomycetemcomitans* with diverse virulence potential have been identified [22, 23] and the complete genome sequences of 38 isolates are currently deposited at the National Center for Biotechnology (NCBI) database. Earlier studies have shown limited presence of virulence determinants involved in the toxicity and colonization of this bacterium, with from 50% to 80% of isolates carrying the *Aa*CDT operon genes [18, 20, 24]. In contrast, our preliminary studies indicated a high rate of virulence of *A*. *actinomycetemcomitans* isolates among Slovenian patients. We identified *A*. *actinomycetemcomitans* in 57% of patients (n = 30) with moderate or advanced generalized chronic periodontitis, and all of the isolates (although two were uncultivable) harbored genes for leukotoxin (*ltxA*), for the CDT toxin subunits (*cdtA*, *cdtB*, *cdtC*), and for colonization factors Flp-1 and ApaH [25]. Our genotyping of *A*. *actinomycetemcomitans* identified two serotype c isolates with normal *cdtA* and *cdtC*, but an exceptionally shorter *cdtB* gene that was missing 219 nucleotides, in comparison to the standard *cdtB* genes [26] (S1A Fig). This surprising finding prompted us to investigate whether this shorter *cdtB* is translated and whether it is cytotoxic.

Here we show that an *A*. *actinomycetemcomitans* isolate with a regularly organized *cdt* operon that includes *cdtA* and *cdtC* but a shorter *cdtB*, the *A*. *actinomycetemcomitans* DO15 isolate, secretes an aberrant CdtB protein that lacks 73 amino acids out of 283; i.e., CdtBΔ116–188 (henceforth CdtB210). We show that this CdtB210 protein is not toxic to Jurkat or HeLa cells, neither when combined with CdtA and CdtC, nor when ectopically expressed. The roles of this natural shorter CdtB variant for the periodontopathogen and for CDT biology remain to be elucidated.

## Materials and Methods

### Microbiological samples, genetic profiling and culture conditions

*Aggregatibacter actinomycetemcomitans* strains were isolated from plaque samples of 17 adult patients with moderate or advanced chronic periodontitis obtained during a cross-sectional study in Slovenia [26]. Between May 23, 2012, and May 15, 2013, 30 patients were selected consecutively from those referred to the Centre for Oral Diseases and Periodontology, University Medical Centre, Ljubljana. The inclusion criteria were: moderate or severe chronic periodontitis, as defined by [27]; age ≥25 years and <60 years; ≥4 teeth in each quadrant of dentition with at least one site per quadrant with a probing pocket depth ≥5 mm and with bleeding on probing; and radiographic evidence of alveolar bone loss in each quadrant of dentition. Patients were excluded for previous periodontal treatment within the last year, systemic or topical antimicrobial therapy in the four weeks prior to the study, and pregnancy. Each participant provided written consent to participate in the study. Ethical permission for the study and the consent procedure was obtained from the Republic of Slovenia National Medical Ethics Committee—NMEC (No. 48/09/12).

After full-mouth periodontal examinations, plaque samples were collected with paper points, as pooled samples from sites selected according to the clinical and radiographic records. The sites with the maximum clinical attachment loss in each quadrant of the dentition were selected (total, four sites). After careful removal of supragingival plaque deposits and isolation of the sampling sites using cotton rolls and gentle air-drying, two sterile absorbent paper points (size #30, Maillefer, Ballaigues, Switzerland) were inserted consecutively into the depth of the pocket and left in place for 10 s. The paper points from all four of the selected periodontal sites were pooled in 1.5 mL reduced transport fluid (0.45 g/L K_2_HPO_4_, 0.45 g/L KH_2_PO_4_, 0.90 g/L NaCl, 90 g/L (NH_4_)_2_SO_4_, 0.18 g/L MgSO_4_, 0.38 g/L EDTA, 0.40 g/L Na_2_CO_3_, 0.20 g/L dithiothreitol) [28].

The plaque samples were grown on selective Dentaid-1 medium [26, 29] in air with 5% CO_2_ at 35°C for 72 h, and one *A*. *actinomycetemcomitans* isolate was identified per individual according to colony morphologies, positive catalase reactions, and MALDI-TOF mass spectrometry (MS) using Biotyper Microflex LT and the MALDI Biotyper 3.0 software (Bruker Daltonik, Bremen, Germany). Genetic profiling for specific virulent factors and serotype characterization was performed by PCR ([26], see S1 Table), using synthetic oligonucleotide primers (S2 Table) as described previously [17, 30].

Smooth isogenic strains of the *A*. *actinomycetemcomitans* isolates used for the secretome preparation were obtained as reported previously [31]. The *E*. *coli* DH5α strain was routinely used for the cloning procedures. The *E*. *coli* BL21(DE3) strain without or with pLysE was used for CdtA and CdtB protein expression, while the *E*. *coli* Rosetta(DE3) strain with pLysS was used for CdtC expression. All of the *E*. *coli* strains were grown in lysogeny broth at 37°C.

### Plasmid construction

*Aggregatibacter actinomycetemcomitans* genomic DNA was obtained with Genomic DNA Purification kits (Thermo Fisher Scientific Inc., U.S.A.). The synthetic oligonucleotide primer pairs shown in S2 Table in the Supporting Information were used to amplify the components of the *cdt* operon. PCR amplification was performed using Vent^®^ polymerase, according to the manufacturer instructions (New England Biolabs, U.K.).

For the protein purification, the amplified genes encoding the CDT proteins without the signal sequences [32] were cloned into the pET21c(+) bacterial expression vector (Novagen; Merck, U.S.A.), using NdeI and XhoI. For the eukaryotic expression, the genes coding for CdtB and CdtB210 were cloned into the pmCherry-C1 mammalian expression vector (Clontech, U.S.A.) as the mCherry C-terminal fusion proteins, using the XhoI and BamHI sites. The sequence coding for the Flag-tag was added at the C-terminus of these two plasmids. Plasmid constructs were routinely validated by sequencing (Macrogen, The Netherlands).

### Secretome preparation and mass-spectrometry analysis

The *A*. *actinomycetemcomitans* strains were streaked on plates with trypticase soy agar (Sigma-Aldrich, Germany) and grown in an anarobic atmosphere without shaking for 48 h at 37°C. The bacteria were further inoculated into 10 mL trypticase soy broth until the stationary phase of growth was achieved, when the 2% inoculum was transferred into 50-mL fresh trypticase soy broth and grown for a further 18 h. The bacteria were removed by centrifugation (6,000× *g*, 15 min, 4°C), and the supernatant was filtered through 0.22-μm-pore-size membranes. The supernatant was cooled, and trichloroacetic acid (Sigma-Aldrich, Germany) was slowly added, to a final concentration of 15% (w/v); this was then left mixing for 3 h at 4°C. Protein aggregates were collected by centrifugation, washed twice with cold acetone, and dried under nitrogen gas. The pellet was resuspended in NuPAGE^®^ LDS Sample Buffer (Thermo Fisher Scientific Inc., U.S.A.) and frozen at -80°C.

The secretome sample was resolved on 12% sodium dodecyl sulfate (SDS)-PAGE gels (Invitrogen, U.S.A.) and visualized using imidazole-SDS-Zn^2+^ reverse staining [33]. To identify the different proteins in the SDS-PAGE gels, the protein bands in the 15 kDa to 35 kDa range were excised from the gel and destained in 70% (v/v) Tris/glycine (50 mM/0.3 M) and 30% (v/v) acetonitrile (ACN), and then sequentially washed in 10 mM NH_4_HCO_3_, 10 mM NH_4_HCO_3_/ ACN (1:1, v/v), and 100% ACN. The proteins were incubated in a reducing buffer (containing 10 mM dithiothreitol in 25 mM NH_4_HCO_3_) for 45 min at 56°C. The reduced proteins were alkylated for 30 min in the dark with 55 mM iodoacetamide in the same buffer without dithiothreitol. Gel slices were subsequently dried with ACN and digested with MS-grade modified trypsin (Promega, U.S.A.) in 25 mM NH_4_HCO_3_ at 37°C overnight. The resulting peptides were extracted with 50% (v/v) ACN/ 5% (v/v) formic acid, concentrated under vacuum to 20 μL, and stored at −20°C. The peptides from each slice were purified on C18 StageTips (Thermo Fisher Scientific Inc., U.S.A.), according to the manufacturer instructions, and analyzed using electrospray ionization ion trap MS (MSD Trap XCT Plus, Agilent), as described previously [33]. The MS and tandem MS/MS spectra were searched against sequences of CDT subunits and the NCBI non-redundant database, using the Spectrum Mill MS Proteomics software (Agilent Technologies, CA, U.S.A.). The Scaffold version 2_06_02 software (Proteome Software Inc., U.S.A.) was used for semi-quantitative analysis of the CdtB proteins identified.

### Purification of recombinant CDT proteins

*Escherichia coli* Rosetta (DE3) pLysS containing the *cdtC* gene fusion, and *E*. *coli* BL21(DE3) carrying one of the other recombinant genes (S2 Table), were grown at 37°C with shaking in 500-mL lysogeny broth containing 100 μg/mL ampicillin and 35 μg/mL chloramphenicol, or only 100 μg/mL ampicillin, respectively. Protein expression was induced at an OD_600_ of 0.8 by addition of 0.5 mM IPTG. After 4 h to 5 h at 37°C, the cells were collected by centrifugation (6,000× *g*, 20 min, 4°C) and stored at -20°C. The recombinant proteins were purified from inclusion bodies, as previously described [34]. The washed inclusion bodies were resuspended in binding buffer (20 mM Tris-HCl, pH 8.0, 500 mM NaCl, 5 mM imidazole, 6 M guanidinium hydrochloride, 5 mM ß-mercaptoethanol), and stirred for 3 h at 4°C. The homogenate was centrifuged at 30,000× *g* for 30 min at 4°C, and 1 mL of HIS-Select^®^ HF Nickel Affinity Gel beads (Sigma-Aldrich, Germany) was added to the supernatant (25 mL). Following a 1-h incubated at 4°C, the beads containing the bound proteins were washed thoroughly, first with binding buffer and then with washing buffer (20 mM Tris-HCl, pH 8.0, 500 mM NaCl, 10 mM imidazole, 8 M urea). Proteins were eluted with elution buffer (20 mM Tris-HCl, pH 8.0, 500 mM NaCl, 300 mM imidazole, 8 M urea) and sequentially dialyzed at 4°C against refolding buffer (100 mM Tris-HCl, pH 7.5, 400 mM L-arginine, 20% [v/v] glycerol) without urea or with 5 M, 3 M or 1.5 M urea, changing the buffer every 24 h. For some experiments, the CdtB protein was additionally purified by cation-exchange chromatography on a Mono S column (GE Healthcare, U.S.A.). The protein was eluted by increasing the salt concentration from 0.0 M to 0.4 M NaCl in the mobile phase (20 mM MES, pH 6.5, 5% [v/v] glycerol). For the enzyme assays, the proteins were eluted through a PD-10 (pre-equilibrated) desalting column (GE Healthcare, U.S.A.), using 20 mM Tris-HCl, pH 7.9, 100 mM NaCl, 10% (v/v) glycerol as elution buffer, and then concentrated using Amicon^®^ Ultra-15 10-kDa molecular-weight cut-off filters (Millipore, U.S.A.). Protein concentrations were determined using Pierce™ BCA Protein Assay kits, using bovine serum albumin as the standard (Thermo Fisher Scientific Inc., U.S.A.).

### Western blotting

To analyze the purified proteins, aliquots of the protein samples were mixed with NuPAGE^®^ LDS Sample Buffer (Thermo Fisher Scientific Inc., U.S.A.), and for the reducing condition, dithiothreitol was added to a final concentration of 50 mM. The amount of protein in the total cell lysates was adjusted to 25 μg per well in SDS electrophoresis sample buffer [35].

Prior to loading onto the gels, the samples were incubated at 95°C for 10 min. The fractionated proteins were visualized with Coomassie blue or were transferred to poly(vinylidene fluoride) membranes (Millipore, U.S.A.), with Western blotting at 150 mA for 90 min. The membranes were blocked in wash buffer (10 mM Tris-HCl, pH 7.4, 50 mM NaCl, 0.025% Tween 20) containing 3% (w/v) bovine serum albumin. The membranes were incubated for 2 h with mouse anti-penta-histidine antibodies (Quiagen, Germany) for anti-His staining, with rabbit antiphospho-H2AX (Cell Signaling Technology, U.S.A.) for γH2AX detection or with mouse anti-Flag (Sigma-Aldrich, Germany) for Flag-tagged protein detection. Appropriate horseradish-peroxidase-labeled secondary antibodies were used according to the manufacturer instructions (GE Healthcare, U.S.A.). Blots for anti-His staining were developed with 4-chloro-1-naphthol solution (Sigma-Aldrich, Germany), and the other blots were developed with enhanced chemiluminescence (ECL; Thermo Scientific Inc., U.S.A.).

### DNase assay

The DNase activities were determined as described previously by Elwell and Dreyfus [11]. The supercoiled pUC19 plasmid (0.5 μg per reaction) was incubated with various amounts of Ni-NTA purified CDT proteins in a DNase buffer (25 mM HEPES, pH 7.0, 10 mM MgCl_2_, 0.5 mM CaCl_2_) for 2 h at 37°C. The reactions were stopped by addition of 10 mM EDTA, 6% (v/v) glycerol, and bromophenol blue. For the control, plasmid DNA was incubated in DNase buffer for 2 h at 37°C. The samples were loaded onto 0.8% agarose gels (pre-mixed with ethidium bromide), and subjected to electrophoresis in TBE buffer for 1.5 h at 90 V. Visualization was achieved with a G:BOX (Syngene, U.K.).

### Phosphatase assay

Phosphatase activity was assessed by monitoring dephosphorylation of phosphatidylinositol 3,4,5-trisphosphate (PI-3,4,5-P3) similarly as described before [13, 36]. Reaction mixture (25 μL) consisted of 100 mM Tris (pH 8.0), 10 mM DTT, 200 μM PI-3,4,5-P3 (diC8; Echelon, U.S.A.) and varied amounts of CdtB or CdtB210. Human recombinant PTEN (Cayman Chemicals, U.S.A.) was used as a control enzyme hydrolyzing PI-3,4,5-P3. Hydrolysis was carried out at 37°C for 30 min. Reaction was terminated by the addition of 15 μL of 100 mM N-ethylmaleimide (Sigma-Aldrich). Inorganic phosphate levels were then measured using Malachite green solution (Malachite Green Assay Kit; Echelon); 35 μL of reaction mixture was combined with 100 μL of the malachite green solution for 20 min at room temperature. Absorbance at 620 nm was measured and phosphate release was quantified by comparison to inorganic phosphate standards (Echelon, U.S.A.). Data are means ±SEM of triplicates.

### Oligomerization of the CDT proteins

Analysis of the CDT complexes was performed by size-exclusion chromatography on a Superdex 200 10/300 GL column (GE Healthcare, U.S.A.) equilibrated with 100 mM Tris-HCl, pH 7.5, 0.4 M L-arginine, 10% (v/v) glycerol. Each individual CDT subunit was either loaded separately or pre-incubated with the other two subunits at equimolar concentrations for 1 h at room temperature prior to gel filtration. The protein molecular masses were estimated using the standards of aprotinin (6.5 kDa), cytochrome C (12.4 kDa), carbonic anhydrase (29 kDa), bovine serum albumin (66 kDa) and blue dextran (2,000 kDa) (Sigma-Aldrich, Germany). The flow was 0.45 mL/min, and eight 0.5-mL fractions were collected and analyzed by SDS-PAGE and Coomassie blue staining, as described above.

### Cytotoxicity assays

The E6.1 human leukemic Jurkat cell line (ECACC 88042803) was used to determine the cytotoxicity of the CDT holotoxin and its individual components, as previously described [37]. Jurkat cells were maintained in RPMI 1640 supplemented with 10% (v/v) fetal calf serum. Thirty thousand cells were seeded in 100-μL growth medium in 96-well tissue-culture plates. The cells were exposed to medium (control) or equimolar mixtures of the indicated CDT subunits, and incubated for up to 48 h. Cell viability was assessed using the PrestoBlue^®^ Cell Viability Reagent, as described by the manufacturer (Thermo Fisher Scientific Inc., U.S.A.). Fluorescence was measured using a plate reader with 570–12 and 615–10 excitation and emission filters respectively. Data are means ±SEM of two independent experiments, each carried out in duplicate. The difference in cytotoxicity was assessed using parallel-line assay analysis (PLA 1.2) software (Stegmann Systems, Germany).

### Cell transfection

HeLa cells were maintained in RPMI 1640 supplemented with 10% (v/v) fetal calf serum. Briefly, 100,000 cells were seeded on 13-mm-diameter slides (Merck, Germany) in 12-well plates with 2 mL medium/well. The following day, the cells were either infected with bacteria or used for transfections with the plasmid encoding the mCherry or mCherry-CdtB fusion proteins (S2 Table) using the jetPEI^®^ reagent, according to the manufacturer instructions (Polyplus transfection, France). Twenty four hours post-transfection, immunocytochemistry staining for γH2AX was performed, and the proteins were imaged using fluorescence microscopy. Two independent experiments were performed.

### Effect of *A*. *actinomycetemcomitans* strains and their spent culture supernatants on HeLa cells

The *A*. *actinomycetemcomitans* D7S [38], D7S *ΔcdtB* knockout strain [39] and DO15 strain (carrying the gene for CdtB210) were grown for 48 h in trypticase soy broth (Sigma-Aldrich, Germany). Calculated volumes of bacteria cultures were resuspended (based on OD_600_) in RPMI 1640 medium so that a multiplicity of infection factor of 1:100 (HeLa cells: bacteria) was achieved. For the cell treatment with bacterial supernatant, the bacteria cultures were centrifuged for 5 min at 6,000× *g*, and 0.5 mL of the supernatant was filtered through 0.22-μm pore size membranes, added to each well to a final volume of 2 mL RPMI 1640 medium, and inspected after 24 h under optical microscopy.

### Microscopy

After 24 h, the cell morphologies of the infected and supernatant-treated HeLa cells were examined. Cell morphology images were taken under 20× objective magnification with a digital camera (DXM1200F; Nikon). The cells grown for 24 h on glass slides were used for fluorescence microscopy. Adherent cells were washed with phosphate-buffered saline (PBS) and then fixed in 4% formaldehyde for 20 min at room temperature. The slides were washed twice in PBS, blocked in blocking solution (3% bovine serum albumin, 0.02% Tween 20 in PBS) for 30 min at room temperature, and incubated for 1 h at room temperature with rabbit anti-phospho-H2AX antibodies (Cell Signaling Technology, U.S.A.; diluted 1:500 in blocking solution). The slides were washed three times for 5 min in PBS, and then incubated with donkey Alexa Fluor^®^ 488 or goat Alexa Fluor^®^ 568 (Thermo Fisher Scientific Inc., U.S.A.) conjugated anti-rabbit antibodies (diluted 1:1000 in blocking solution) for 1 h at room temperature. Nuclei were counterstained with Vectashield^®^ DAPI solution (Vector Laboratories, U.K.). The slides were mounted and viewed under fluorescence microscopy. Fluorescent images were taken using a monochrome digital camera (Hamamatsu, Germany) using FITC or TRITC filter sets, according to the experiment set-up. All of the pictures were taken under 63x objective magnification using the same settings for each filter, and were then analyzed using the ImageJ software.

### Structural and bioinformatics analyses

Amino-acid sequences were aligned using the on-line ClustalW. The three-dimensional structure model for CdtB210 was obtained using the I-TASSER on-line server [40], and CdtB from *A*. *actinomycetemcomitans* (PDB 2f2f, chain B) was used as the template for homology modeling [41]. The three-dimensional structure alignments of CdtB and CdtB210 were rendered using CHIMERA [42].

The draft genome of the *A*. *actinomycetemcomitans* DO15 isolate was obtained by NGS Illumina 2× 250 bp MiSeq sequencing, provided by Seqmatic (U.S.A.). Contigs were aligned to the genome of the serotype c D11S-1 strain (GeneBank accession number: CP001733.1) [43] by the sequence provider. The genome structure of the new strain was compared to the D11S-1 strain using the MAUVE program [44]. According to the draft genome sequence, the primers (S2 Table) were designed to PCR amplify and re-sequence the *cdt* operon and the flanking region of the *A*. *actinomycetemcomitans* DO15 strain.

## Results and Discussion

### Identification of the *cdt* operon carrying the unique *cdtB*

We isolated *A*. *actinomycetemcomitans* from 17 out of 30 patients with moderate or advanced generalized chronic periodontitis, and performed genetic screening for the virulence factors, leukotoxin (*ltxA*), CDT toxin subunits (*cdtA*, *cdtB*, *cdtC*), and for colonization factors Flp-1 and ApaH (S1 Table). Surprisingly, the aberrant *cdtB* gene (*cdtB210*) was detected in the two of the isolates (S1A Fig). The draft genome sequence (GeneBank accession number: SRP070158) was obtained for one of the *A*. *actinomycetemcomitans* isolates (DO15) that carried the *cdtB* variant gene *cdtB210* (S1 Fig). Although, substantial genomic variations among *A*. *actinomycetemcomitans* strains have been reported [22], the draft genomic sequence of the DO15 isolate showed high identity to the D11S-1 strain (GeneBank accession number: CP001733.1).

**Fig 1:**
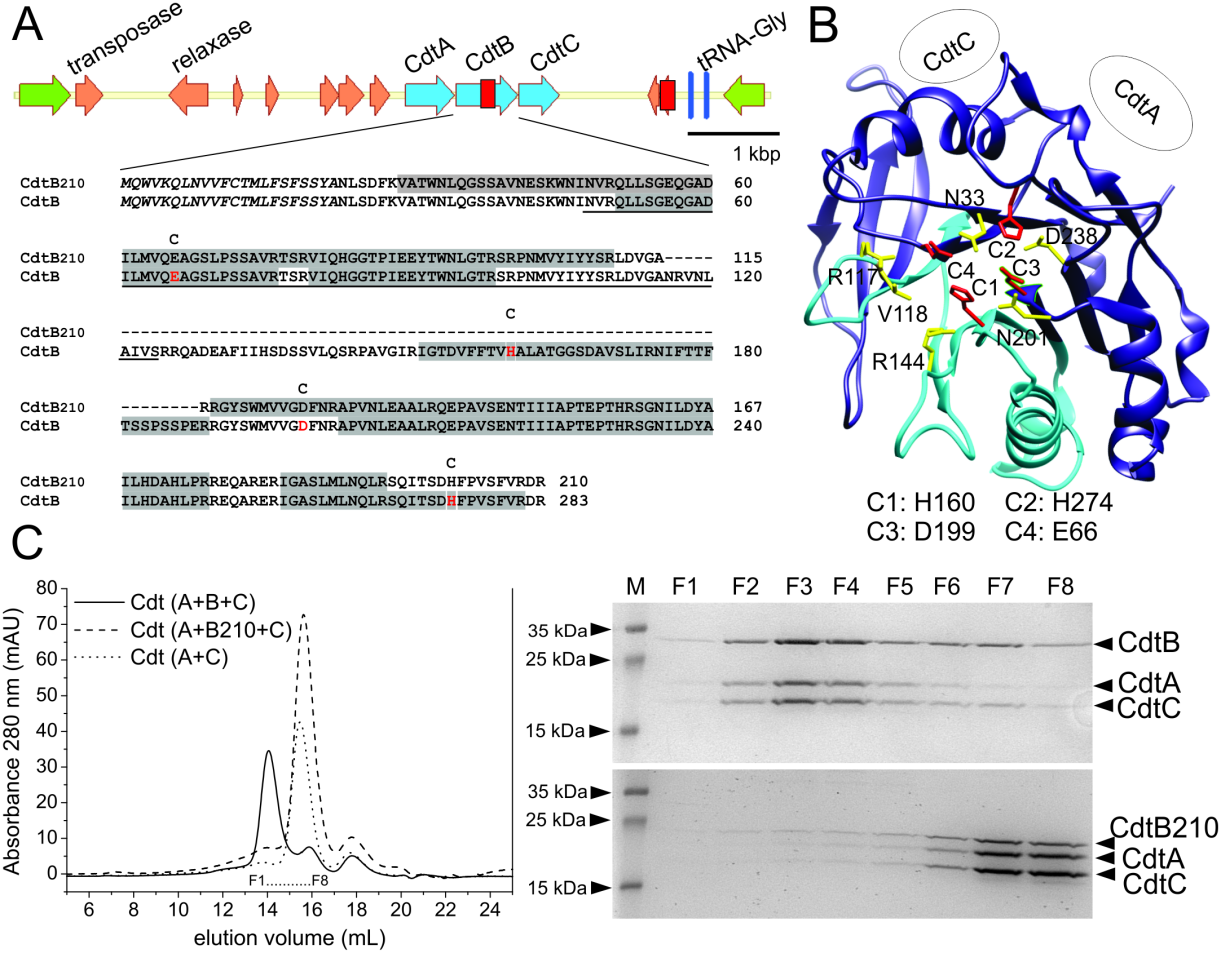
CdtB210 lacks the critical catalytic residue H160, and does not form a holotoxin. (A) Genetic map of the genomic island of the *A*. *actinomycetemcomitans* isolate DO15 that harbors *cdtB210* with the aligned CdtB amino-acid sequences expanded. Flanking genes, chromosomally encoded genes (green), genomic island genes (orange), and *cdt* operon determinants (blue) are marked. The two red boxes designate the regions absent on genomic island of the DO15 strain. First red box: 219 bp missing in *cdtB210*. The deletion includes part of the nuclear localization signal (underlined) and the catalytic H160, critical for DNase activity. The H160, D199, E66 and H274 involved in catalysis are shown (red). The first 22 amino-acid residues in italics designate the signal sequence. The shaded residues were identified by mass spectrometry peptide mapping. Peptides spanning the deletion were identified only in the secretome of the CdtB-producing strain. (B) Structure of *A*. *actinomycetemcomitans* CdtB (blue, PDB 2f2f, chain B) with the region missing in CdtB210 (cyan). Residues involved in catalysis (red sticks, and at the bottom) and substrate/ metal binding (yellow sticks) are shown. The contacts between the CdtB, CdtA, and CdtC proteins in the holotoxin are presented schematically. (C) Analysis of the oligomeric state of CdtB and CdtB210 premixed (equimolar) with CdtA and CdtC. CdtA plus CdtC was used as the control. Molecular mass analysis of the CDT complexes was performed on a Superdex 200 column. F1 to F8: fractions assayed using SDS-PAGE analysis (shown right), which indicated that CdtB, but not CdtB210, formed the tripartite complex.

Alignment of the D11S-1 *cdt* operon sequence demonstrated that the *cdt* gene cluster is encoded on a genomic island [22], and as for *cdt*, this carries the remnants of heterologous mobile genetic element sequences, which suggests horizontal origins (Fig 1A). The most notable difference between the DO15 and D11S-1 strains was a 219-bp in-frame-deletion in the *cdtB* gene of *A*. *actinomycetemcomitans* DO15, which results in the CdtB210 protein (Fig 1A and 1B). The natural CdtB210 variant also thus lacks the conserved catalytic H160 residue, the substrate-binding residues R117 and R144 [45], and part of the nuclear localization signal [10, 11] (Fig 1A and 1B). Therefore, these data suggested that this CdtB210 variant might not be genotoxic in eukaryotes.

### The *A*. *actinomycetemcomitans* DO15 isolate secretes the CdtB variant

*Aggregatibacter actinomycetemcomitans* strains excrete CDT into the extracellular environment either via a secretion system [46] or by packing of the toxin into outer-membrane vesicles for delivery into eukaryotic cells [47]. To determine whether CdtB210 is expressed and secreted by the periodontitis-associated *A*. *actinomycetemcomitans* isolates, MS peptide mapping was applied to the extracellular proteome of the isolates grown in plankton (S1B Fig). The peptides identified covered 71% of the CdtB210 sequence (Fig 1A). As the positive control, an *A*. *actinomycetemcomitans* serotype c strain D11S-1 was used. Peptide mapping confirmed the presence of full-length CdtB in the secretome of this serotype c strain (Fig 1A, S3 and S4 Tables). Semi-quantitative analysis of the MS/MS identified proteins revealed that both CdtB and CdtB210 were produced at similar levels (S5 Table) in their respective *A*. *actinomycetemcomitans* strains. Thus, we demonstrated here that the deleted variant of the *cdtB* gene was expressed by the *A*. *actinomycetemcomitans* DO15 isolate, and thus this genomic part is not a pseudogene.

### CdtB variant analysis

To characterize the biological activity of CdtB210, we cloned and purified recombinant carboxyterminal hexa-histidine tagged CdtA, CdtC, CdtB and CdtB210, which lacked the signal peptide sequences present in the preproteins (S2 Fig). Structural analysis based on the crystal structure of canonical *A*. *actinomycetemcomitans* CDT [41] indicated that the residues missing in the CdtB210 protein do not contribute to the oligomerization interface. This suggested that CdtB210 still forms a heterotrimeric complex with CdtA and CdtC (Fig 1B). However, size-exclusion chromatography of full-length CdtB or CdtB210 premixed with CdtA and CdtC defined the molecular weights of 52.7 kDa and 25.9 kDa, respectively (Fig 1C; S3 Fig). Thus, these data strongly imply that CdtB210 fails to form a stable tripartite complex with CdtA and CdtC, and consequently it will not enter the targeted cells.

Compared to full-length CdtB, CdtB210 lacks several residues that are involved in phosphodiesterase activity (Fig 1B), and therefore we predicted that the CdtB210 had lost the CtdB DNase activity. To test this, *in-vitro* DNase assays were performed using the pUC19 plasmid as substrate, which was incubated with purified full-length CdtB or CdtB210. The nicked, or linearized form of the plasmid increased when it was incubated with full-length CdtB, which was paralleled by a decrease in the supercoiled form. This indicated that, as expected, full-length CdtB has DNA-nicking activity (S4A Fig). Surprisingly, comparable, or even higher activity was seen for CdtB210 (S4A Fig). However, a similar degradation pattern was also observed when the pUC19 plasmid was incubated with the CdtA or CdtC protein (S4B Fig). In the presence of the chelator EDTA, CdtB did not show nicking activity (S4B Fig). In addition to the standard CDT isolation protocol [34], we therefore applied an ion-exchange chromatography step to remove potential DNase contaminants from the Ni-NTA purified CdtB protein. This additional purification (S4C and S4D Fig) reduced the DNase activity of CdtB (S4E Fig) but did not markedly affect its cytotoxicity (S4F Fig). Collectively, the observed divalent-cation-dependent DNase activities of CdtB and CdtB210, the DNase activities of CdtA and CdtC, and the decreased nicking activity of CdtB purified by ion-exchange chromatography strongly suggest that the observed DNase activity of the recombinant CDT proteins is due to minor contaminants that originated from the hosting *E*. *coli* strain. Furthermore, we tested CdtB and CdtB210 for phosphatidylinositol 3,4,5-trisphosphate phosphatase activity as reported in [13], however, neither of the variants was active in the 30-min assay (S5 Fig). In parallel with reports that have challenged the role of the *A*. *actinomycetemcomitans* CdtB DNase activity in its cell cytotoxicity [13, 48, 49], our observation indeed calls for further re-examination of the phosphatase activities of CDTs.

### The *A*. *actinomycetemcomitans* DO15 isolate and purified CdtB210 are not genotoxic

**Fig 2:**
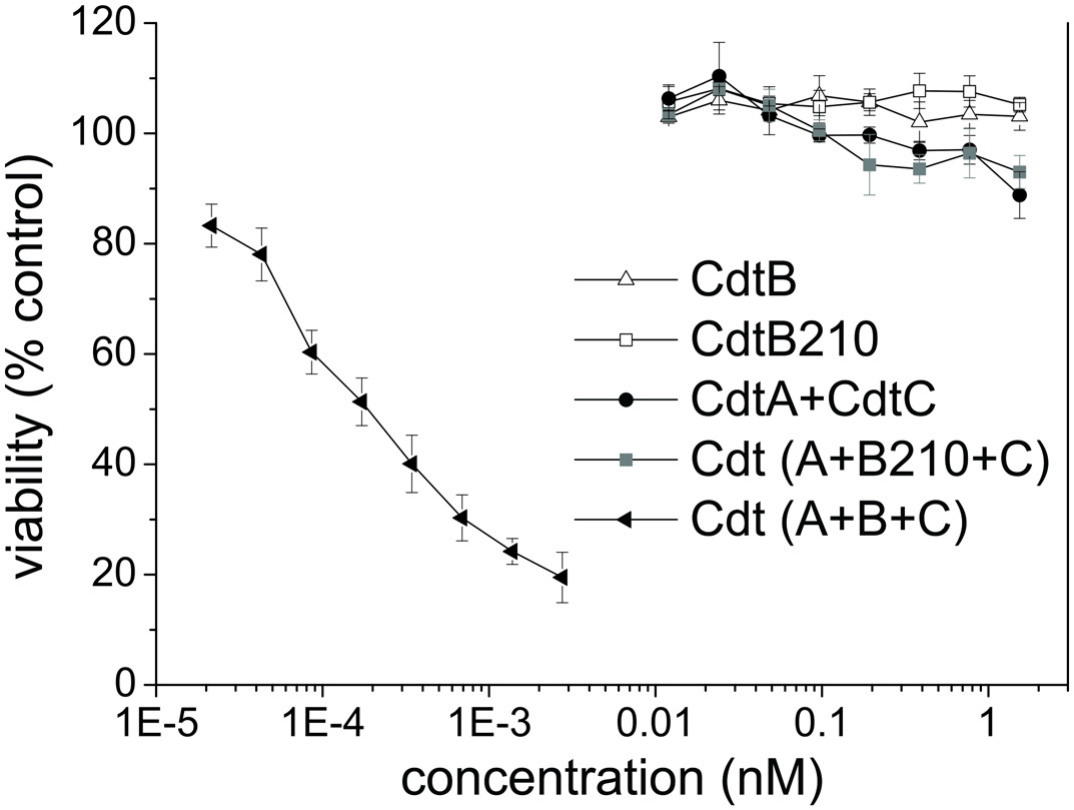
CdtB210 does not intoxicate eukaryotic cells. Cell toxicity assay of equimolar mixtures of the purified CDT subunits (as indicated) in the Jurkat cell line after 48 h incubation as described in Methods. Sub-picomolar toxic effects were only detected for the holotoxin with CdtB. Data are average of two independent experiments presented with standard error, each carried out in duplicate.

We examined whether CdtB210 can intoxicate eukaryotic cells. Jurkat cells were treated with the prepared *Aa*CDT subunits. The CDT holoenzyme with full-length CdtB was toxic for the Jurkat cells in the sub-picomolar range, as expected (Fig 2), and as reported previously [50]. In contrast, either alone or combined with CdtA and CdtC, CdtB210 was not cytotoxic (Fig 2).

**Fig 3:**
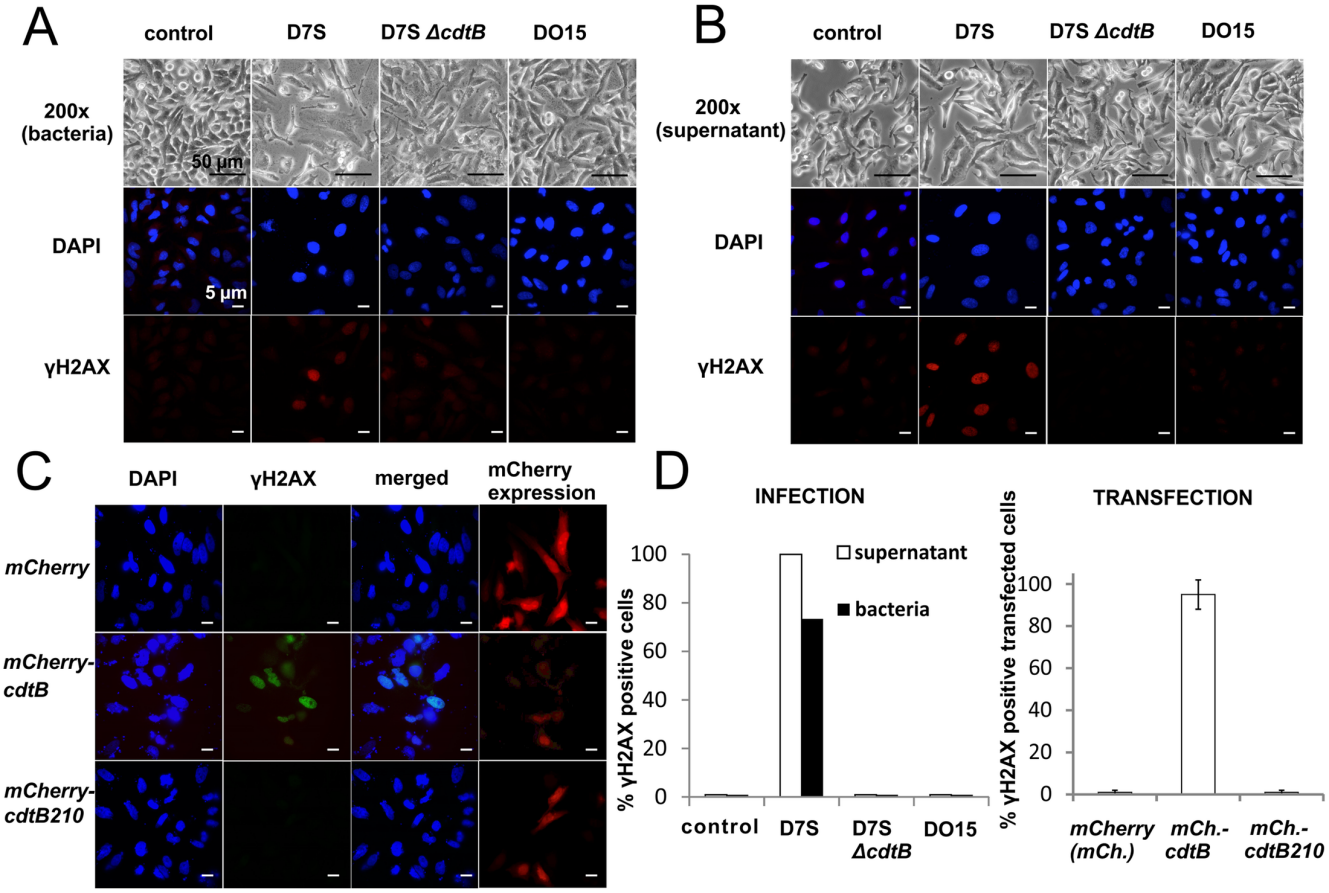
The *A*. *actinomycetemcomitans* DO15 isolate and its gene product CdtB210 are not genotoxic to eukaryotic cells. (A, B) The *A*. *actinomycetemcomitans* strains D7S, its isogenic knockout D7S ΔcdtB, and DO15 (A) or their supernatants (B) were incubated with HeLa cells. Effects on the HeLa morphology and immunocytochemistry staining for γH2AX were monitored 24 h post-infection. Cellular distention of HeLa was evident by infection with the D7S strain coding for CdtB, while the DO15 isolate harboring CdtB210 did not cause cellular distention or H2AX phosphorylation. Cell medium was used for the control. (C) Transfection of HeLa cells was performed as described in Materials and Methods. Genotoxic effects (i.e., staining for γH2AX) were observed only in cells transfected with the plasmid coding for mCherry-CdtB. (D) Quantification of the infected HeLa cells (n = 100) and transfected (two experiments with standard error, n = 90) HeLa cells, as in (A, B) and (C), respectively. Cells with more than 5 foci of γH2AX were considered positive. Genotoxicity was only seen for the D7S strain and for CdtB.

Although CdtB210 cannot be internalized into eukaryotic cells due to its inability to form a tripartite complex (Fig 1C and 1D), to determine whether it can induce damage of nuclear DNA, CdtB210 was expressed in HeLa cells. The cells were transfected with a eukaryotic expression vector encoding the genes for either CdtB or CdtB210 as mCherry C-terminal fusion partners. These data show that ectopic expression of CdtB210 did not induce H2AX phosphorylation (Fig 3C and 3D, S6 Fig), which is a marker for DNA damage [51, 52] and has already been well documented in the context of CDT intoxication [12, 53, 54]. Our photomicrographs showed that both mCherry-CdtB and mCherry-CdtB210 entered the nucleus (Fig 3C), most probably either by diffusion [55] or by active transport [10]. However, intracellular mCherry-CdtB was genotoxic, but mCherry-CdtB210 was not. In addition, we also determined whether the DO15 isolate harboring *cdtB210* can elicit DNA damage. We hypothesized that this strain might produce factors to assist CdtB210 to affect eukaryotic cells. To test this, we infected HeLa cells with the DO15 isolate or treated the cells with the filter-sterilized supernatant of the isolate. Here, neither cytoplasmic distention, which is a typical morphological marker of CDT intoxication [14], nor H2AX phosphorylation of the cells was observed (Fig 3A, 3B and 3D). These results were supported by the toxic activity of the D7S strain but not of its deletion mutant, the Δ*cdtB* variant strain, which were used as positive and negative controls, respectively. Thus, here we provide evidence that the *A*. *actinomycetemcomitans* DO15 isolate that secretes the CdtB210 variant cannot intoxicate HeLa or Jurkat cells.

In conclusion, we have isolated an *A*. *actinomycetemcomitans* DO15 strain that produces the regular CdtA and CdtC proteins, but produces the aberrant CdtB210. This shorter CdtB is not able to form the tripartite CDT and thus cannot be translocated into eukaryotic cells. It is also not cytotoxic when delivered intracellularly. Further studies are required to define the biological role and the relevance of this novel CdtB variant, CdtB210, in the context of *A*. *actinomycetemcomitans* infections. This will in turn provide a tool to obtain better insight into the roles and mechanisms of action of *A*. *actinomycetemcomitans* and other CDTs.

## Supporting Information

S1 Fig Genetic analysis and secretome preparation of CdtB and CdtB210. (A) Representative gel following PCR using the standard *cdtB* primers [3], performed on genomic DNA isolated from 15 *A*. *actinomycetemcomitans* strains isolated from Slovenian patients diagnosed with chronic periodontitis. Two of these isolated strains harbored a *cdtB* gene with an in-frame deletion (*cdtB210*). (B) Representative SDS-PAGE of the secretome preparations (mass spectrometry: bands from 15–35 kDa) obtained from *A*. *actinomycetemcomitans* strains harboring either the *cdtB* or *cdtB210* genes (as indicated). M, molecular mass standards.
(TIF)

S2 Fig Purification of the His_6_-tagged recombinant CDT subunits. (A) Representative SDS-PAGE of the recombinantly expressed CdtA, CdtB, CdtB210 and CdtC proteins (as indicated), under reducing/ non-reducing conditions, stained with Coomassie blue. (B) Representative Western blot of the purified proteins using an antibody specific for the His-tag (see Materials and Methods). M, molecular mass standards.
(TIF)

S3 Fig Size-exclusion chromatography of the individual CDT proteins and protein standards. (A) Individual CDT subunits run on a Superdex 200 column (10/300 GL) showing the elution peaks (as indicated). (B) Protein standards on Superdex 200 column to determine molecular mass of the CDT complexes.
(TIF)

S4 Fig DNase assay of CDT proteins on the pUC19 plasmid, and effects of additional ion-exchange chromatography purification on the cytotoxicity of CdtB. (A, B) Representative agarose gel electrophoresis for DNase activities of CdtB and CdtB210 incubated with plasmid pUC19 (see Materials and Methods) (A), and of individual CDT subunits A, B, B210, and C (B). CdtB210 appears to have higher DNAse activity than CdtB (A). Addition of EDTA abolishes DNA degradation by CdtB and CdtB210 (B). OC, open circular; L, linear; SC, supercoiled DNA. (C) Representative ion-exchange chromatography on a Mono S column of Ni-NTA pre-purified CdtB. F1 to F6 indicate the collected fractions. (D) Representative SDS-PAGE of Ni-NTA (IEC load) pre-elution fraction and fractions F1 to F6 from (C). M, molecular mass standards. (E) Representative agarose gel electrophoresis for the DNase activities of CdtB during purification. Activity of Ni-NTA pre-elution fraction of CdtB (IEC load) is greater (>5-fold) that of CdtB from fraction F4 (and F5) from the ion-exchange chromatography (IEC; see (D)). OC, open circular; L, linear; SC, supercoiled DNA. (F) Cytotoxicity assay on the Jurkat cell line (see Methods) of CdtB before (Ni-NTA, IEC load) and after the ion-exchange chromatography (fraction 4, IEC), supplemented with CdtA plus CdtC at equimolar concentrations. Data are average of two independent experiments presented with standard error, each carried out in duplicate. The cytotoxicity of CdtB after the ion-exchange chromatography purification drops by ~17% (see Cytotoxicity assay, Materials and Methods).
(TIF)

S5 Fig *A*. *actinomycetemcomitans* CdtB and CdtB210 do not exhibit phosphatidylinositol 3,4,5-trisphosphate phosphatase activity. Phosphatase activity was assessed by monitoring the dephosphorylation of PI-3,4,5-P3 (30 min at room temperature) using Malachite green solution. Human recombinant PTEN was used as a positive control. Data is represented as mean ±SEM of triplicate.
(TIF)

S6 Fig Ectopic expression of the CdtB210 protein does not induce DNA damage. Representative Western blotting 24 h post-transfection from HeLa cells transfected with plasmids encoding the indicated proteins, to determine the expression of the proteins and H2AX phosphorylation. The fusion CdtB proteins were C-terminal Flag-tagged.
(TIF)

S1 Table Prevalence of virulence factors in *A*. *actinomycetemcomitans* strains isolated from Slovenian patients with periodontitis. (DOCX)

S2 Table Primers and plasmids used in this study. (DOCX)

S3 Table CDT peptides detected by mass spectrometry in the secretome of the *A*. *actinomycetemcomitans* wild-type strain. (DOCX)

S4 Table CDT peptides detected by mass spectrometry in the secretome of the *A*. *actinomycetemcomitans* DO15 isolate. (DOCX)

S5 Table Semi-quantitative analysis of CdtB proteins from mass spectrometry. (DOCX)
