# Supplementary figures and images for: A Cytolethal Distending Toxin Variant from Aggregatibacter actinomycetemcomitans with an Aberrant CdtB That Lacks the Conserved Catalytic Histidine 160
Source: PLoS One. 2016 Jul 14;11(7):e0159231. doi: 10.1371/journal.pone.0159231 (PMC4945079; doi:10.1371/journal.pone.0159231)

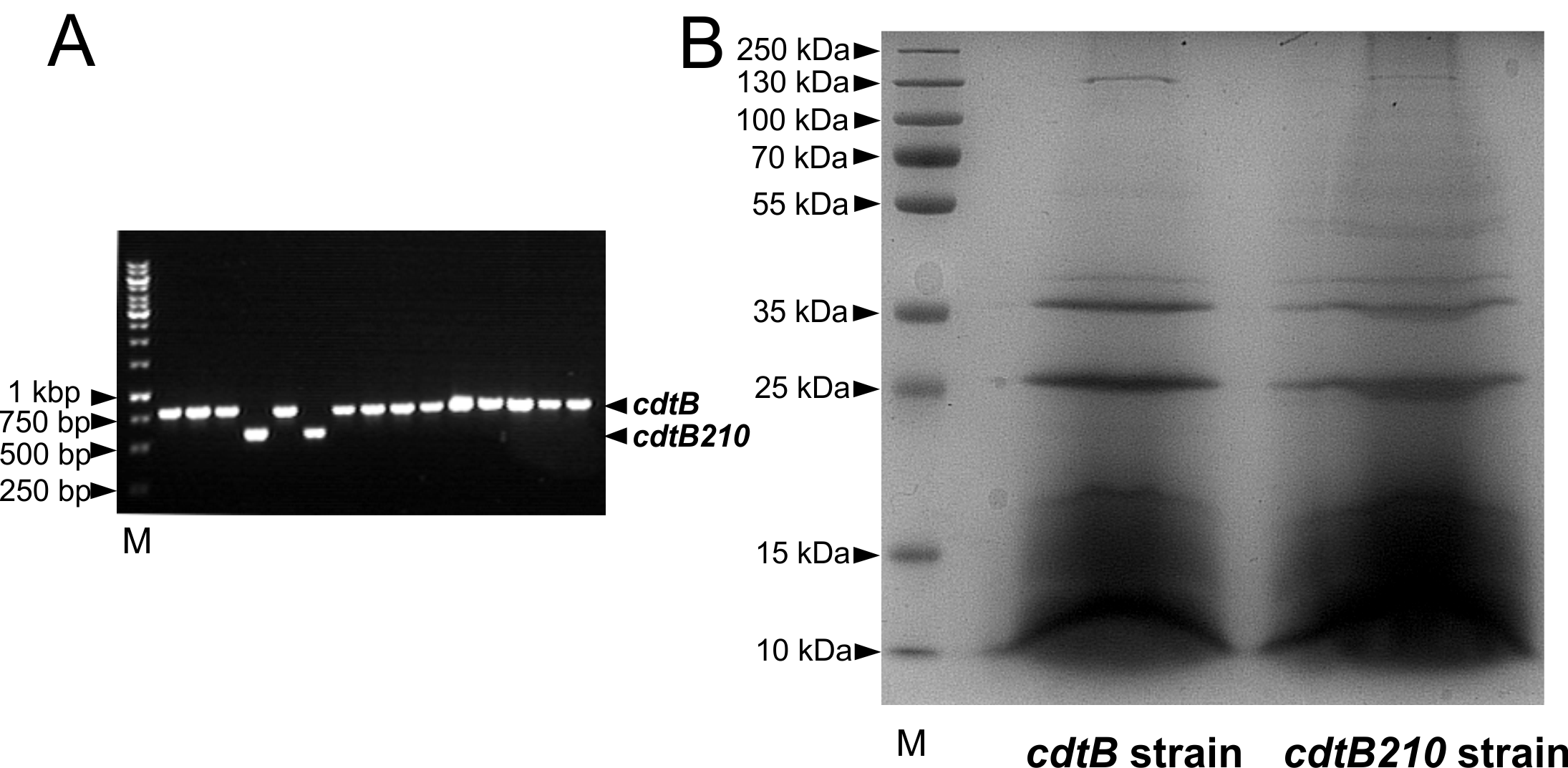

Supplement: S1 Fig — (A) Representative gel following PCR using the standard cdtB primers [3], performed on genomic DNA isolated from 15 A. actinomycetemcomitans strains isolated from Slovenian patients diagnosed with chronic periodontitis. Two of these isolated strains harbored a cdtB gene with an in-frame deletion (cdtB210). (B) Representative SDS-PAGE of the secretome preparations (mass spectrometry: bands from 15–35 kDa) obtained from A. actinomycetemcomitans strains harboring either the cdtB or cdtB210 genes (as indicated). M, molecular mass standards. (TIF) [file pone.0159231.s001.tif]

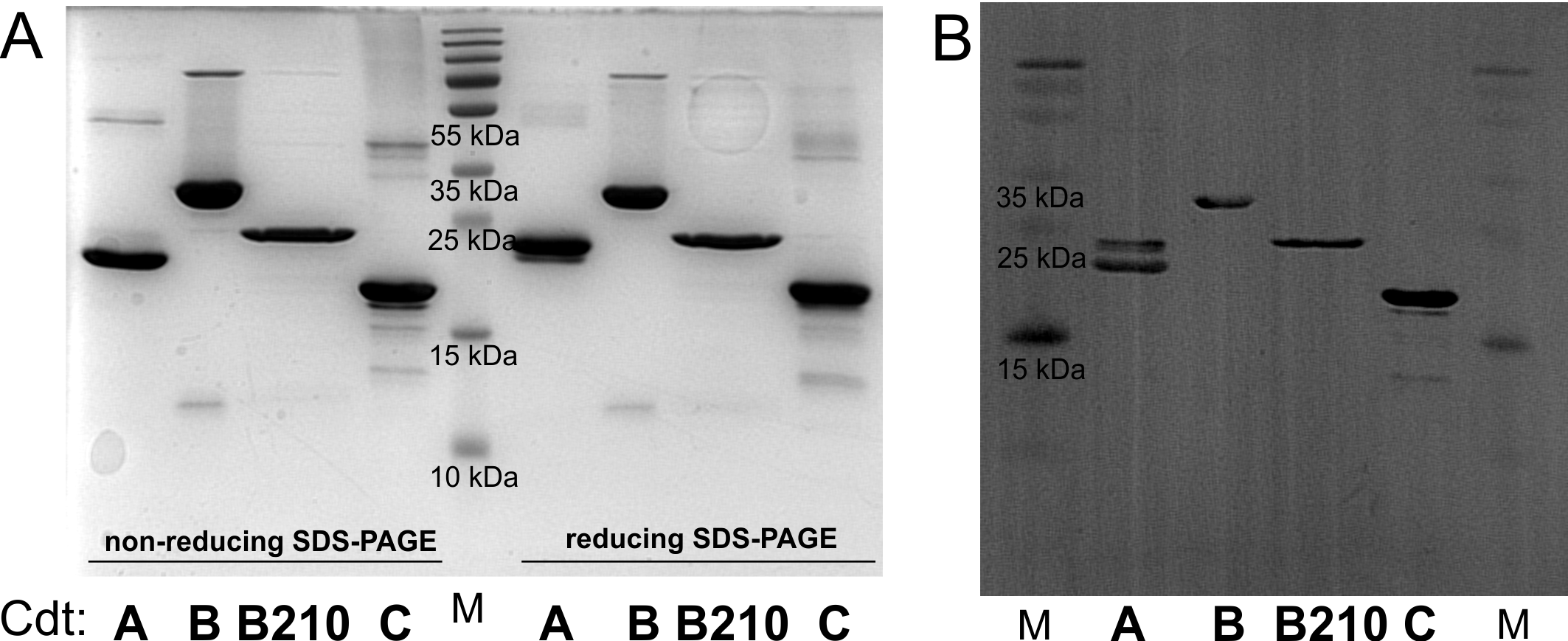

Supplement: S2 Fig — (A) Representative SDS-PAGE of the recombinantly expressed CdtA, CdtB, CdtB210 and CdtC proteins (as indicated), under reducing/ non-reducing conditions, stained with Coomassie blue. (B) Representative Western blot of the purified proteins using an antibody specific for the His-tag (see Materials and Methods). M, molecular mass standards. (TIF) [file pone.0159231.s002.tif]

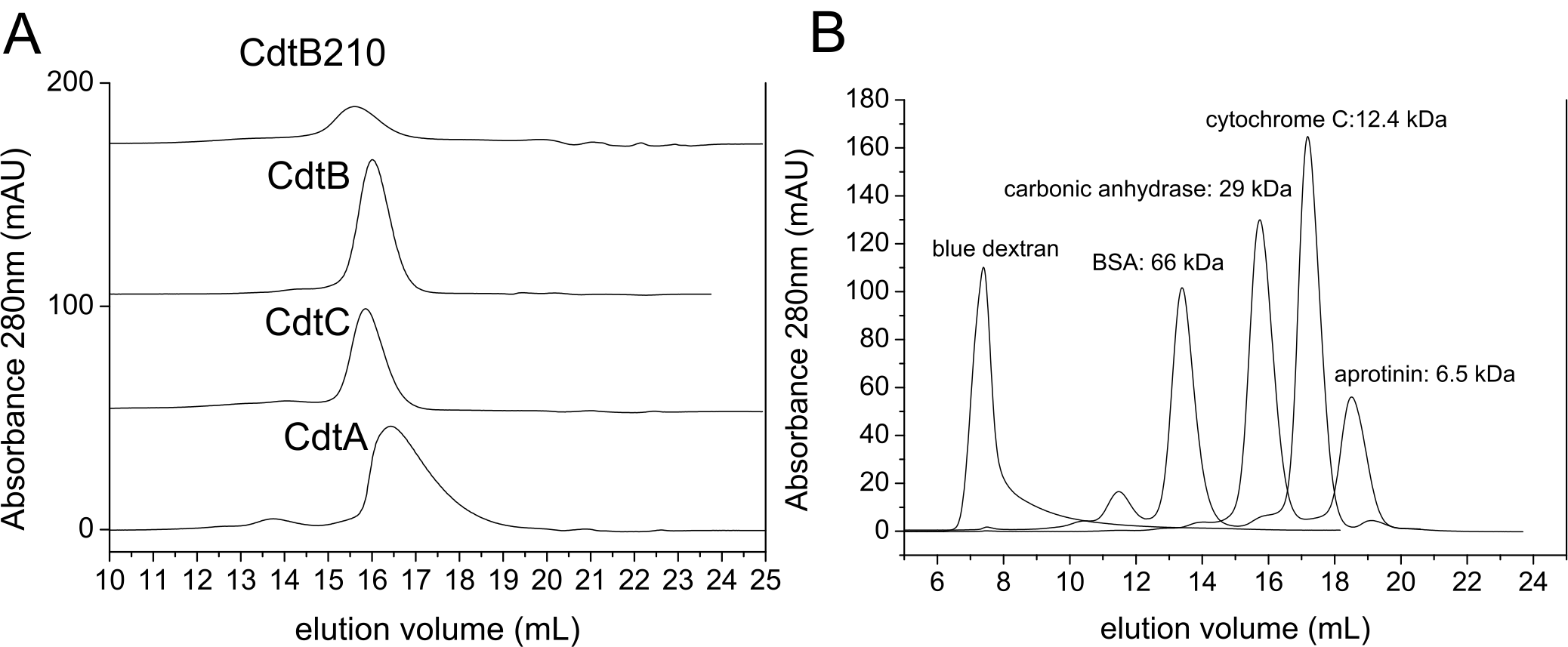

Supplement: S3 Fig — (A) Individual CDT subunits run on a Superdex 200 column (10/300 GL) showing the elution peaks (as indicated). (B) Protein standards on Superdex 200 column to determine molecular mass of the CDT complexes. (TIF) [file pone.0159231.s003.tif]

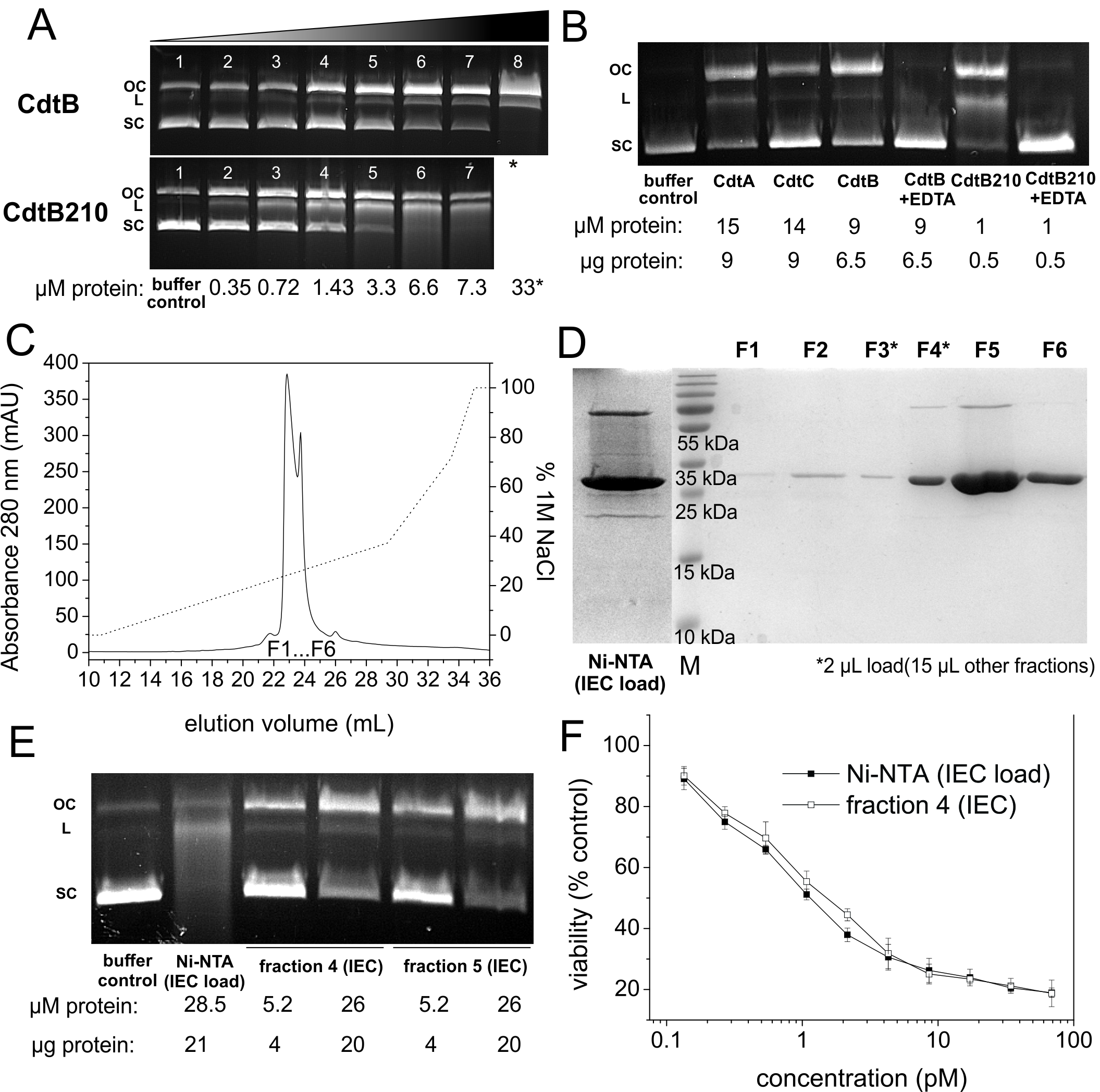

Supplement: S4 Fig — (A, B) Representative agarose gel electrophoresis for DNase activities of CdtB and CdtB210 incubated with plasmid pUC19 (see Materials and Methods) (A), and of individual CDT subunits A, B, B210, and C (B). CdtB210 appears to have higher DNAse activity than CdtB (A). Addition of EDTA abolishes DNA degradation by CdtB and CdtB210 (B). OC, open circular; L, linear; SC, supercoiled DNA. (C) Representative ion-exchange chromatography on a Mono S column of Ni-NTA pre-purified CdtB. F1 to F6 indicate the collected fractions. (D) Representative SDS-PAGE of Ni-NTA (IEC load) pre-elution fraction and fractions F1 to F6 from (C). M, molecular mass standards. (E) Representative agarose gel electrophoresis for the DNase activities of CdtB during purification. Activity of Ni-NTA pre-elution fraction of CdtB (IEC load) is greater (>5-fold) that of CdtB from fraction F4 (and F5) from the ion-exchange chromatography (IEC; see (D)). OC, open circular; L, linear; SC, supercoiled DNA. (F) Cytotoxicity assay on the Jurkat cell line (see Methods) of CdtB before (Ni-NTA, IEC load) and after the ion-exchange chromatography (fraction 4, IEC), supplemented with CdtA plus CdtC at equimolar concentrations. Data are average of two independent experiments presented with standard error, each carried out in duplicate. The cytotoxicity of CdtB after the ion-exchange chromatography purification drops by ~17% (see Cytotoxicity assay, Materials and Methods). (TIF) [file pone.0159231.s004.tif]

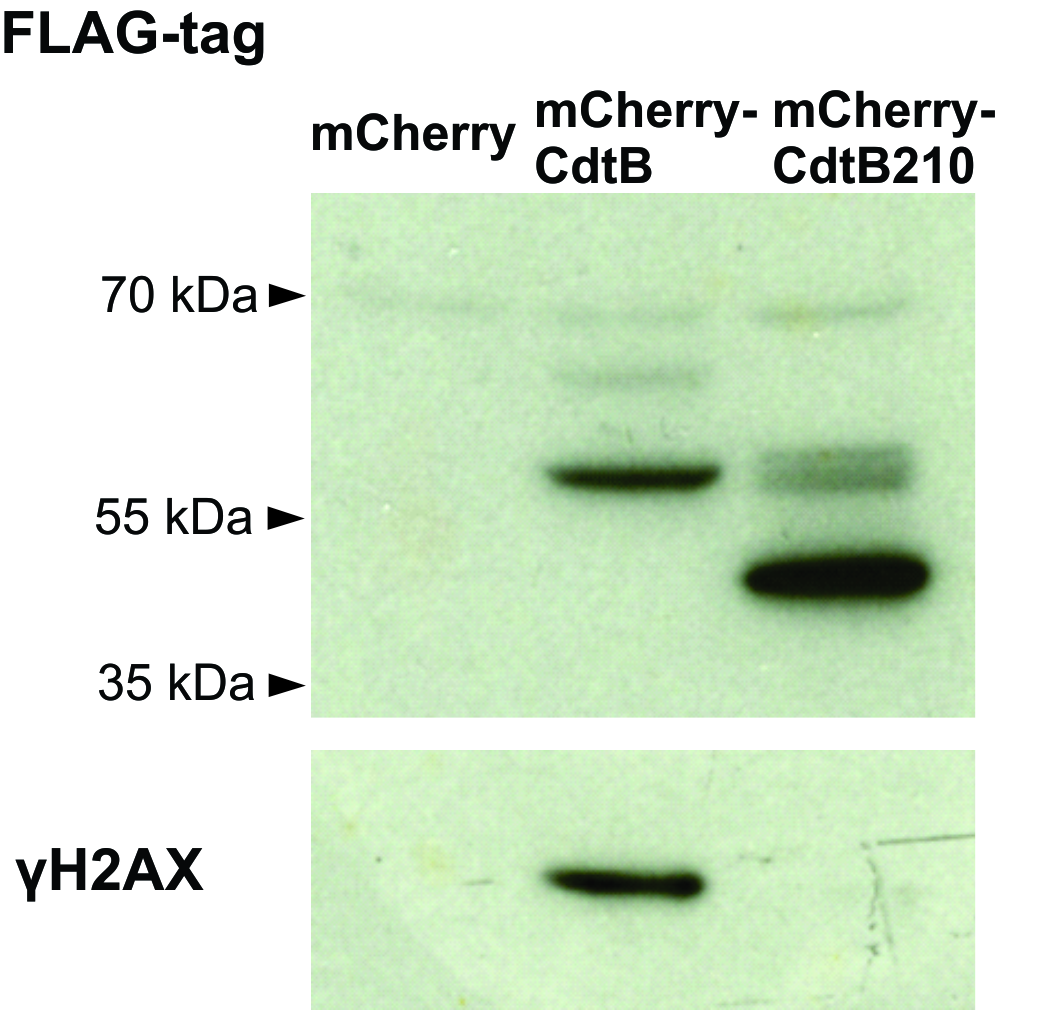

Supplement: S6 Fig — Representative Western blotting 24 h post-transfection from HeLa cells transfected with plasmids encoding the indicated proteins, to determine the expression of the proteins and H2AX phosphorylation. The fusion CdtB proteins were C-terminal Flag-tagged. (TIF) [file pone.0159231.s006.tif]
