# Supplementary material for: A Cytolethal Distending Toxin Variant from Aggregatibacter actinomycetemcomitans with an Aberrant CdtB That Lacks the Conserved Catalytic Histidine 160
Source: PLoS One. 2016 Jul 14;11(7):e0159231. doi: 10.1371/journal.pone.0159231 (PMC4945079; doi:10.1371/journal.pone.0159231)
Supplement: S1 Table — (DOCX) [file pone.0159231.s007.docx]

**S1 Table**. Prevalence of virulence factors in *A. actinomycetemcomitans* strains isolated from Slovenian patients with periodontitis (adapted from [26]).

(*ltxA*= leukotoxin; *apaH*= diadenosine tetraphosphatase; *flp-1*= fimbriae associated protein; *cdtA, cdtB, cdtC*= cytolethal distending toxin subunits A, B and C)

| **Patient\gene** | ***ltxA*** | ***apaH*** | ***flp-1*** | ***cdtA*** | ***cdtB*** | ***cdtC*** |
| --- | --- | --- | --- | --- | --- | --- |
| 1 | **+** | **+** | **+** | **+** | **+** | **+** |
| 2 | **+** | **+** | **+** | **+** | **+** | **+** |
| 3 | **+** | **+** | **+** | **+** | **+** | **+** |
| 4 | **+** | **+** | **+** | **+** | ***cdtB210*** | **+** |
| 9 | **+** | **-** | **+** | **+** | **+** | **+** |
| 16 | **+** | **-** | **+** | **+** | ***cdtB210*** | **+** |
| 18 | **+** | **+** | **+** | **+** | **+** | **+** |
| 20 | **+** | **+** | **+** | **+** | **+** | **+** |
| 22 | **+** | **+** | **+** | **+** | **+** | **+** |
| 25 | **+** | **+** | **+** | **+** | **+** | **+** |
| 31 | **+** | **+** | **+** | **+** | **+** | **+** |
| 32 | **+** | **+** | **+** | **+** | **+** | **+** |
| 34 | **+** | **+** | **+** | **+** | **+** | **+** |
| 35 | **+** | **+** | **+** | **+** | **+** | **+** |
| 36 | **+** | **+** | **+** | **+** | **+** | **+** |

**+** indicates presence of PCR amplicon

*oligonucleotide primers used for PCR amplification are listed in S2 Table
