# Supplementary material for: A Cytolethal Distending Toxin Variant from Aggregatibacter actinomycetemcomitans with an Aberrant CdtB That Lacks the Conserved Catalytic Histidine 160
Source: PLoS One. 2016 Jul 14;11(7):e0159231. doi: 10.1371/journal.pone.0159231 (PMC4945079; doi:10.1371/journal.pone.0159231)
Supplement: S2 Table — (DOCX) [file pone.0159231.s008.docx]

**S2 Table:** **Primers and plasmids used in this study.**

| **Plasmid/ primer** | | **Primer (5´ to 3´)**  **(restriction sites underlined)** | **Description** |
| --- | --- | --- | --- |
| pET21cCdtA | | GCGCATATGAGTGACTATTCTCAGCCTGAA | CdtA-His6 encoded in pET21c+ plasmid (Ap^R^) |
|  |  | GCCCTCGAGATTAACCGCTGTTGCTTCTAA |  |
| pET21cCdtB | | GCGCATATGAACTTGAGTGATTTCAAAGTA | CdtB-His6 encoded in pET21c+ plasmid (Ap^R^) |
|  |  | GCCCTCGAGGCGATCACGAACAAAACTAAC |  |
| pET21cCdtB210 | | GCGCATATGAACTTGAGTGATTTCAAAGTA | CdtB210-His6 encoded in pET21c+ plasmid (Ap^R^) |
|  |  | GCCCTCGAGGCGATCACGAACAAAACTAAC |  |
| pET21cCdtC | | GCGCATATGGAATCAAATCCTGATCCGACT | CdtC-His6 encoded in pET21c+ plasmid (Ap^R^) |
|  |  | GCCCTCGAGGCTACCCTGATTTCTCCCCAC |  |
| pC1CdtB_Fusion | | GCGCTCGAGCTAACTTGAGTGATTTCAAAGTA | mCherry-CdtB-Flag fusion protein encoded in pmCherry-C1 plasmid (Kan^R^) |
|  |  | GCGGGATCCTTACTTGTCATCGTCGTCCTTGTAGTCGCGATCACGAACAAAACTAACA |  |
| pC1CdtB210_Fusion | | GCGCTCGAGCTAACTTGAGTGATTTCAAAGTA | mCherry-CdtB210-Flag fusion protein encoded in pmCherry-C1 plasmid (Kan^R^) |
|  |  | GCGGGATCCTTACTTGTCATCGTCGTCCTTGTAGTCGCGATCACGAACAAAACTAACA |  |
| *ltxA* | Forward | TCGCGAATCAGCTCGCCG | 285 bp PCR amplicon* |
|  | Reverse | GCTTTGCAAGCTCCTCACC |  |
| *flp1* | Forward | AACAACAATAGGAGCATTAAGACA | 300 bp PCR amplicon* |
|  | Reverse | GTATTTAATATTTAAGTTGTTACTTATT |  |
| *apaH* | Forward | ATTTAATCGGCGACCTGCAC | 825 bp PCR amplicon* |
|  | Reverse | TGTCTTCCCAACGTAGCATG |  |
| *cdtA* | Forward | GGTTTAGTGGCTTGT | 583 bp PCR amplicon* |
|  | Reverse | CACGTAATGGTTCTGTT |  |
| *cdtB* | Forward | GGTTTTCTGTACGATGT | 790 (571 bp for *cdtB210*) bp PCR amplicon* |
|  | Reverse | GGATGTAATTTGTGAGCGT |  |
| *cdtC* | Forward | GACTTTGACGAGTCATGCA | 512 bp PCR amplicon* |
|  | Reverse | CCTGATTTCTCCCCA |  |
| 1 | Forward | TTAGATGTATTGATCTTTAAT | 1,400 bp PCR fragment |
|  | Reverse | TTGAGTAACGCCCCTCAGCAA |  |
| 2 | Forward | TCATTGGGAGCGGAGCATTTA | 1,050 bp PCR fragment |
|  | Reverse | AACCAAACAATGTCGTTACGT |  |
| 3 | Forward | TGAAGCTGATTTATTAAAAGG | 1,050 bp PCR fragment |
|  | Reverse | CGTTGATTTGACGAACAAGCC |  |
| 4 | Forward | AGGATAAAATAAGAAAAATTT | 1,060 bp PCR fragment |
|  | Reverse | CATCTAAACGGGAATAATAAA |  |
| 5 | Forward | CGGCAGTAAGAACCTCACGAG | 1,175 bp PCR fragment* (deletion in *cdtB*) |
|  | Reverse | TGCCACCGGACATAAAGATCG |  |
| 6 | Forward | GACAGGAAGTAAATGACACAG | 1,150 bp PCR fragment |
|  | Reverse | ATCTATTGAGTTAGTTTTTGC |  |
| 7 | Forward | AAGTAGCTCTATCTTATCTTT | 820 bp PCR fragment** (deletion of a hypothetical protein, GenBank: ACX82307.1) |
|  | Reverse | CTGATCATAACTTAAATTTGA |  |

* reference in the main text

**PCR fragments that confirm deletion in a contig or between contigs of the *cdt* genomic island (see Fig. 1A).
