# Supplementary material for: A Cytolethal Distending Toxin Variant from Aggregatibacter actinomycetemcomitans with an Aberrant CdtB That Lacks the Conserved Catalytic Histidine 160
Source: PLoS One. 2016 Jul 14;11(7):e0159231. doi: 10.1371/journal.pone.0159231 (PMC4945079; doi:10.1371/journal.pone.0159231)
Supplement: S3 Table — (DOCX) [file pone.0159231.s009.docx]

**S3 Table.** CDT peptides detected by mass-spectrometry in the secretome of the *A. actinomycetemcomitans* wild-type strain.

| **Protein** | **Spectra N^o^** | **Distinct peptides N^o^** | **Distinct summed MS/MS search score** | **% Coverage** | **Mean peptide spectral intensity** | **MS/MS-derived sequence** | ***m/z* measured [Da]** | **z** | **MH+ matched [Da]** | **Spectrum intensity** |
| --- | --- | --- | --- | --- | --- | --- | --- | --- | --- | --- |
| CdtB | 10 | 9 | 139.02 | 55 | 8.73E+07 | (R)IGTDVFFTVHALATGGSDAVSLIR(N) | 816.88 | 3 | 2447.29 | 1.88E+07 |
|  |  |  |  |  |  | (R)NIFTTFTSSPSSPER(R) | 836.14 | 2 | 1670.80 | 6.75E+07 |
|  |  |  |  |  |  | (R)QLLSGEQGADILMVQEAGSLPSSAVR(T) | 886.53 | 3 | 2656.36 | 1.30E+08 |
|  |  |  |  |  |  | (R)QEPAVSENTIIIAPTEPTHR(S) | 735.38 | 3 | 2203.13 | 6.04E+07 |
|  |  |  |  |  |  | (R)VIQHGGTPIEEYTWNLGTR(S) | 724.73 | 3 | 2171.08 | 1.81E+08 |
|  |  |  |  |  |  | (R)SGNILDYAILHDAHLPR(R) | 635.90 | 3 | 1904.99 | 1.04E+08 |
|  |  |  |  |  |  | (R)APVNLEAALR(Q) | 527.81 | 2 | 1053.60 | 3.03E+07 |
|  |  |  |  |  |  | (R)IGASLMLNQLR(S) | 608.67 | 2 | 1215.68 | 9.40E+07 |
|  |  |  |  |  |  | (R)IGASLMLNQLR(S) | 608.62 | 2 | 1215.68 | 9.51E+07 |
|  |  |  |  |  |  | (R)SQITSDHFPVSFVR(D) | 541.06 | 3 | 1619.81 | 9,20E+07 |
| CdtA | 2 | 2 | 33.45 | 14.00 | 8.80e+07 | (K)LAQEFELLPTDSGAVVIK(S) | 965.77 | 2 | 1930.05 | 8.79e+07 |
|  |  |  |  |  |  | (K)SSTTQFQPQPLLSK(A) | 781.53 | 2 | 1561.82 | 8.81e+07 |
| CdtC | 3 | 3 | 37.85 | 26 | 1.22e+07 | (K)DIDHTVFNLIPTNTGAFLIK(D) | 744.23 | 3 | 2229.19 | 1.06e+07 |
|  |  |  |  |  |  | (R)LEPCGISVSGR(T) | 588.18 | 2 | 1174.59 | 6.72e+06 |
|  |  |  |  |  |  | (R)TFSLAYQWGILPPFGPSK(I) | 1005.82 | 2 | 2009,05 | 1.94e+07 |
