# Supplementary material for: A Cytolethal Distending Toxin Variant from Aggregatibacter actinomycetemcomitans with an Aberrant CdtB That Lacks the Conserved Catalytic Histidine 160
Source: PLoS One. 2016 Jul 14;11(7):e0159231. doi: 10.1371/journal.pone.0159231 (PMC4945079; doi:10.1371/journal.pone.0159231)
Supplement: S4 Table — (DOCX) [file pone.0159231.s010.docx]

**S4 Table.** CDT peptides detected by mass spectrometry in the secretome of the *A. actinomycetemcomitans* DO15 isolate.

| **Protein** | **Spectra No** | **Distinct peptides No** | **Distinct summed MS/MS search score** | **% Coverage** | **Mean peptide spectral intensity** | **MS/MS-derived sequence** | ***m/z* measured [Da]** | **z** | **MH+ matched [Da]** | **Spectrum intensity** |
| --- | --- | --- | --- | --- | --- | --- | --- | --- | --- | --- |
| CdtB210 | 13 | 10 | 195.65 | 71 | 4.32e+08 | (R)APVNLEAALR(Q) | 527.54 | 2 | 1053.61 | 2.57e+08 |
|  |  |  |  |  |  | (R)GYSWMVVGDFNR(A) | 715.93 | 2 | 1430.65 | 1.96e+08 |
|  |  |  |  |  |  | (R)IGASLMLNQLR(S) | 608.62 | 2 | 1215.69 | 7.62e+08 |
|  |  |  |  |  |  | (R)QEPAVSENTIIIAPTEPTHR(S) | 735.39 | 3 | 2203.14 | 3.88e+08 |
|  |  |  |  |  |  | (R)QLLSGEQGADILMVQEAGSLPSSAVR(T) | 886.59 | 3 | 2656.36 | 8.50e+08 |
|  |  |  |  |  |  | (R)QLLSGEQGADILMoxVQEAGSLPSSAVR(T) | 891.88 | 3 | 2656.36 | 2.60e+08 |
|  |  |  |  |  |  | (R)RGYSWMVVGDFNR(A) | 529.66 | 3 | 1586.75 | 9.01e+07 |
|  |  |  |  |  |  | (R)SGNILDYAILHDAHLPR(R) | 635.99 | 3 | 1905.00 | 1.19e+09 |
|  |  |  |  |  |  | (R)SRPNMVYIYYSR(L) | 517.37 | 3 | 1548.76 | 2.46e+07 |
|  |  |  |  |  |  | (K)VATWNLQGSSAVNESK(W) | 846.26 | 2 | 1690.84 | 6.49e+07 |
|  |  |  |  |  |  | (R)VIQHGGTPIEEYTWNLGTR(S) | 724.52 | 3 | 2171.09 | 1.52e+09 |
|  |  |  |  |  |  | (K)WNINVR(Q) | 401.71 | 2 | 801.44 | 9.27e+06 |
| CdtA | 5 | 4 | 65.31 | 27 | 4.85e+07 | (K)ASSMoxPLNLLSSSK(N) | 676.15 | 2 | 1334.70 | 5.94e+07 |
|  |  |  |  |  |  | (K)LAQEFELLPTDSGAVVIK(S) | 965.67 | 2 | 1930.05 | 5.07e+07 |
|  |  |  |  |  |  | (R)MSDYSQPESQSDLAPK(S) | 892.20 | 2 | 1782.79 | 3.75e+07 |
|  |  |  |  |  |  | (R)MoxSDYSQPESQSDLAPK(S) | 900.18 | 2 | 1782.79 | 2.50e+07 |
|  |  |  |  |  |  | (K)SSTTQFQPQPLLSK(A) | 781.68 | 2 | 1561.82 | 7.00e+07 |
