# Supplementary material for: A Cytolethal Distending Toxin Variant from Aggregatibacter actinomycetemcomitans with an Aberrant CdtB That Lacks the Conserved Catalytic Histidine 160
Source: PLoS One. 2016 Jul 14;11(7):e0159231. doi: 10.1371/journal.pone.0159231 (PMC4945079; doi:10.1371/journal.pone.0159231)
Supplement: S5 Table — (DOCX) [file pone.0159231.s011.docx]

**S5 Table.** Semi-quantitative analysis of CdtB proteins from mass spectrometry.

| Experiment number | Protein | Mean peptide spectral intensity in samples | Spectra number per sample |
| --- | --- | --- | --- |
| 1 | CdtB | 2.83e+08 | 29 |
|  | CdtB210 | 9.55e+08 | 53 |
| 2 | CdtB | 1.69e+09 | 15 |
|  | CdtB210 | 1.32e+09 | 31 |
